# Novel Fe^II^ and Co^II^ Complexes of Natural Product Tryptanthrin: Synthesis and Binding with G-Quadruplex DNA

**DOI:** 10.1155/2016/5075847

**Published:** 2016-09-06

**Authors:** Yi-ning Zhong, Yan Zhang, Yun-qiong Gu, Shi-yun Wu, Wen-ying Shen, Ming-xiong Tan

**Affiliations:** ^1^College of Pharmacy of Guangxi University of Chinese Medicine, Nanning, Guangxi 530299, China; ^2^Guangxi Key Laboratory for Agricultural Resources Chemistry and Efficient Utilization (Cultivation Base), Yulin Normal University, Yulin, Guangxi 537000, China; ^3^The Key Laboratory for the Chemistry and Molecular Engineering of Medicinal Resources, Guangxi Normal University, Guilin, Guangxi 541004, China

## Abstract

Tryptanthrin is one of the most important members of indoloquinoline alkaloids. We obtained this alkaloid from* Isatis*. Two novel Fe^II^ and Co^II^ complexes of tryptanthrin were first synthesized. Single-crystal X-ray diffraction analyses show that these complexes display distorted four-coordinated tetrahedron geometry via two heterocyclic nitrogen and oxygen atoms from tryptanthrin ligand. Binding with G-quadruplex DNA properties revealed that both complexes were found to exhibit significant interaction with G-quadruplex DNA. This study may potentially serve as the basis of future rational design of metal-based drugs from natural products that target the G-quadruplex DNA.

## 1. Introduction

G-quadruplexes is regarded as four-stranded structures that are made up of guanine (G) bases in a purine-rich DNA duplex [1, 2]. Considerable evidence suggests that these structures are found at the telomeric ends of chromosomes and regulate the expression of several important oncogenes [3, 4]. Based on these observations, G-quadruplexes have been proposed as a potential target for anticancer drug design [5–7]. Detailed investigations have been carried out for human telomeric and c-myc, c-kit, k-ras, and bcl-2 quadruplexes [8–12]. The first example of a small-molecule TMPyP4 was found to reduce the transcriptional activity of a gene (c-myc) with a promoter-G-quadruplex motif as a target [13]. An isoalloxazine small-molecule G-quadruplex ligand that binds both c-kit G-quadruplexes was shown to reduce the levels of c-kit mRNA in c-kit expressing cell line [14]. Studies have also confirmed the presence of a G-quadruplex-forming sequence within the promoter of k-ras [15], which is also sensitive to a reduction in transcriptional activity induced by the G-quadruplex interactive ligand TMPyP4. A zinc(II) isopropylguanidinium-phthalocyanine complex was shown to be of very high affinity and selectivity for c-myc, H-telo, and Kras mutation [16]. Quindoline derivatives show that turning off transcription of the* bcl-2* Gene by stabilizing the bcl-2 promotes quadruplex structure [17]. Metal complexes from alkaloids with a large planar *π*-aromatic conjugated system would be beneficial to increase affinity to the grooves of the quadruplex by *π*-*π* stacking and electrostatic interactions [18–20].

**Figure 1:**
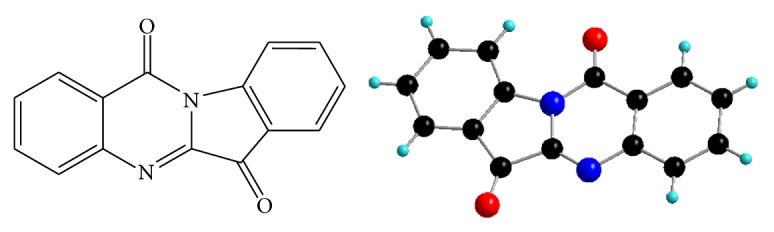
The chemical structure of tryptanthrin.

Tryptanthrin (Figure 1) is one of the most important members of indoloquinoline alkaloids [21]. This alkaloid is found in a number of plants like* Isatis* [22],* Calanthe* [23],* Strobilanthes* [24],* Couroupita* [25], and* Wrightia* [26]. Tryptanthrin exhibits diverse biological effects, such as antimicrobial, antitumor, and anti-inflammatory activities [24, 27, 28]. Tryptanthrin has been used as Chinese medicine and folk medicine for treatment of anti-inflammatory, antipyretic, and analgesic effects [29]. Studies have shown that alkaloids such as cryptolepine [30], berberine [31], liriodenine [32], sanguinarine [33], and nitidine [34] exhibit G-quadruplex strong stabilization activities and structure-dependent interactions. However, relatively less attention has been paid to tryptanthrin and its derivatives that bind to and stabilize G-quadruplex DNA. We conceive that tryptanthrin derivatives would be of interest for G-quadruplex DNA binding due to the planar structure and large *π*-conjugated system.

**Figure 2:**
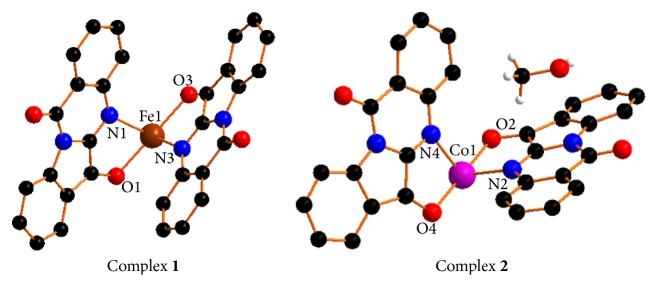
The crystal structures of complexes** 1** and** 2**.

Therefore, we synthesized the first example of metal-mediated natural product tryptanthrin complexes of iron(II) (Figure 2, complex** 1**) and cobalt(II) (Figure 2, complex** 1**) as the G-quadruplex binders and investigated their abilities to act as selective and effective G-quadruplex binders. The approach is based on *π*-conjugation planar of indoloquinoline alkaloids, the remarkable biological effects, and the photophysical, magnetic, or catalytic properties of metal complexes. It would be anticipated that the formation of metal complexes with the planar tryptanthrin ligand does have the potential to stack on or intercalate with guanine of G-quadruplex, and the charged molecules as a whole can bind to the grooves and loops in the negatively charged sugar-phosphate backbone of the DNA.

## 2. Experimental

### 2.1. Materials and Methods

Infrared spectra were obtained on a PerkinElmer FT-IR spectrometer. Fluorescence measurements were performed on a Shimadzu RF-5301/PC spectrofluorophotometer. The X-ray diffraction data were collected on a Bruker Smart Apex II and a Rigaku Saturn CCD diffractometer equipped with graphite monochromated Mo-Ka radiation (*λ* = 0.71074 Å).

All chemical reagents were commercially available and received without further purification, unless noted specifically. Tryptanthrins were isolated from the Chinese plants of* Isatis* according to the literature methods [24]. G-quadruplex DNA HTG21 (5′-GGGTTAGGGTTAGGGTTAGGG-3′, stored at 4°C; long-term storage at –20°C) are obtained from Shanghai Sangon Biological Engineering Technology & Services (Shanghai, China). The DNA concentration per pair was determined based on the absorbance value at *λ* = 260 nm (*ε*_260_ = 3.81 × 10^5^ M (strand)^−1^ cm^−1^) for DNA oligomers by using UV/Vis absorption spectroscopy. Unless otherwise stated, spectroscopic titration experiments were carried out in 10 mM Tris-HCl (pH 7.35) containing 100 mM KCl. All tumor cell lines were obtained from the Shanghai Institute for Biological Science (China).

Stock solutions of all the compounds (2 mM) were made in DMSO. Further dilutions to working concentrations were made with corresponding buffer. The formation of all intramolecular G-quadruplexes was analyzed as follows: the oligomers samples, dissolved in Tris-KCl-HCl buffer, were heated to 95°C for 10 min, gently cooled to room temperature, and then incubated at 4°C overnight. All the spectroscopic experiments were performed at room temperature.

### 2.2. Spectra Characteristics Analysis

Absorption titrations and fluorescence emission titration were performed by using a fixed compounds concentration (2.0 × 10^−3^ M) and varying the concentration of G4-HTG21 (1.0 × 10^−5^ M, 5 *μ*L per scan). While measuring absorption titrations, the solutions were allowed to incubate for 10 min before the spectra were recorded and an equal amount of G4-HTG21 was added to both the compound solution and the reference solution to eliminate the absorbance of G4-HTG21 itself [35, 36]. Fluorescence quenching spectra of ethidium bromide (EthBr) bound with G4-DNA were performed with increasing amounts of complexes** 1** and** 2**, ranging ratios of [complex]/[EthBr] from 0 : 1 to 10 : 1, when the ration of [DNA]/[EthBr] remained 10 : 1, excited at 453 nm. The quenching constant, *K*_SV_, was calculated according to the classic Stern-Volmer equation.

### 2.3. Synthesis of Complexes

#### 2.3.1. Synthesis of [Fe(Try)_2_] (**1**)

**1** was synthesized in a mixture of Try (0.05 mmol, 0.0124 g), FeCl_2_·4H_2_O (0.06 mmol, 0.0119 g), CH_3_OH (1 mL), and DMF (1.5 mL) placed in a thick Pyrex tube. The sealed tube was heated at 100°C for 3 d to yield brown prismatic-shaped crystals. The crystals were washed with ethanol, dried, and stored under vacuum suitable for X-ray diffraction analysis. Yield: 89.6%. Elemental analysis for C_30_H_14_N_4_O_4_Fe: calcd (%). C 65.48, H 2.56, N 10.18; found (%) C 65.11, H 3.00, N 9.83; IR (KBr, cm^−1^): 2929.0, 1685.3, 1594.5, 1498.5, 1441.1, 1349.7, 1233.1, 1188.8, 1062.1, 8874.9, 764.1 cm^−1^.

#### 2.3.2. Synthesis of [Co(Try)_2_] CH_3_OH (**2**)

**2** was synthesized in a mixture of Try (0.05 mmol, 0.0124 g), CoCl_2_·6H_2_O (0.06 mmol, 0.0158 g), CH_3_OH (0.5 mL), and DMF (2 mL) placed in a thick Pyrex tube. The sealed tube was heated at 100°C for 3 d to yield brown prismatic-shaped crystals. The crystals were washed with ethanol, dried, and stored under vacuum suitable for X-ray diffraction analysis. Yield: 89.6%. Elemental analysis for C_31_H_20_N_4_O_5_Co: calcd (%). C 63.38, H 3.43, N 9.54; found (%) C 63.19, H 3.20, N 9.23; IR (KBr, cm^−1^): 3468.0, 1674.0, 1592.0, 1438.0, 1362.0, 1232.0, 1188.0, 744.0, 586.0 cm^−1^.

## 3. Results and Discussion

### 3.1. X-Ray Crystal Structures Analysis

Complexes** 1** and** 2** were prepared via the reaction of Try with FeCl_2_·4H_2_O or CoCl_2_·6H_2_O in the presence of CH_3_OH and DMF under solvothermal conditions. Single-crystal X-ray diffraction analyses for their structure revealed that in each case the metal ion(II) center is coordinated by Try ligand via two heterocyclic nitrogen and oxygen atoms to form a tetrahedron geometry. One of the major differences is that complex 2 contain a CH_3_OH molecule in the unit cell (Figure 2). The X-ray crystal data collection for two complexes is shown in Table 1.

### 3.2. Selectivity for Binding of G-Quadruplex by Spectroscopic Methods

**Figure 3:**
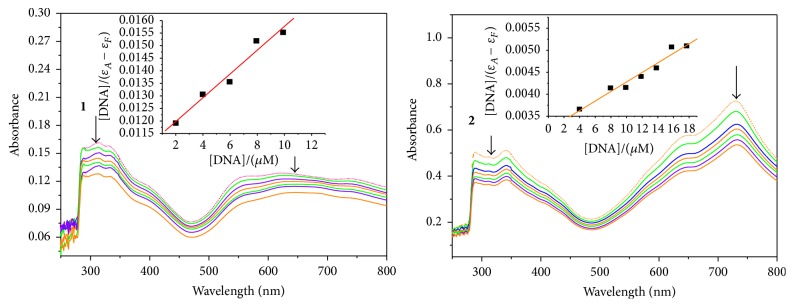
UV-Vis absorption titration of complexes** 1** and** 2** in Tris/KCl buffer (100 mM KCl, 10 mM Tris-HCl, and pH 7.35) with a fixed compound concentration (2.0 × 10^−3^ M) and increasing the amounts of G4-HTG21 (1.0 × 10^−5^ M, 5 *μ*L per scan).

UV-visible absorption titration was performed to determine the binding affinity of the complexes to G-quadruplex. The HTG21-G-quadruplex sample was added sequentially to the complexes of Tris/KCl buffer solutions. UV-vis absorbance spectra were recorded after each addition. As shown in Figure 3, with increasing concentration of HTG21-G-quadruplex, the absorbance at the ligand absorption band region, as well as the MLCT (metal-to-ligand charge transfer) band, decreased with 35% hypochromism at 286 nm for complex** 1** and 35% at 286 nm for complex** 2**. This hypochromic phenomenon is attributed to the strong interaction between the complexes and G-quadruplex DNA.

In order to compare the affinities of the two complexes quantitatively, the Scatchard equation has been applied to evaluate the binding to DNA [37]:

(1) $$\begin{array}{c} \frac{D}{\Delta \varepsilon_{ap}}=\frac{D}{\Delta \varepsilon }+\frac{1}{\left(\Delta \varepsilon +K\right)}. \end{array}$$

Binding constants, *K*, were determined from a reciprocal plot of *D*/Δ*ε*_ap_ versus *D*. In (1), DNA is expressed in base pairs; the apparent molar extinction coefficients *ε*_*A*_ = *A*_obs_/[complex], Δ*ε*_ap_ = |*ε*_*A*_ − *ε*_*F*_|, and Δ*ε* = |*ε*_*B*_ − *ε*_*F*_| with *ε*_*B*_ and *ε*_*F*_ representing the molar extinction coefficients of bound the complex that is intercalated within G-quadruplex and the free complex that is in solution, respectively. The plot of *D*/Δ*ε*_ap_ versus *D* revealed that the binding constant *K* of complexes** 1** and** 2** was 4.29 × 10^4^ dm^3^mol^−1^ and 3.40 × 10^4^ dm^3^mol^−1^, respectively, at 20.0°C (Figure 3 inset).

The binding constant *K* of complex** 1** is larger than that of complex** 2**. It indicated that complex** 1** bound to the DNA more tightly than complex** 2** did. The two complexes have the same intercalative ligand. This is most likely due to the less solubility of complex** 2** than complex** 1** in water at the same condition.

**Figure 4:**
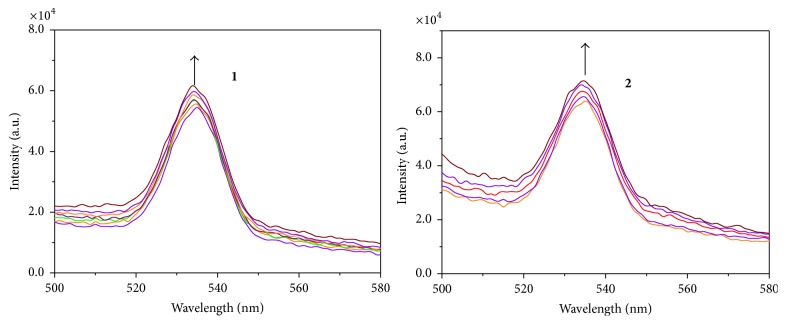
Emission spectra of complexes** 1** and** 2** in Tris/KCl buffer (100 mM KCl, 10 mM Tris-HCl, and pH 7.35) with a fixed compound concentration (2.0 × 10^−3^ M) and varying the amounts of G4-HTG21 (1.0 × 10^−5^ M, 5 *μ*L per scan), excited at 453 nm.

Emission spectral measurements were used to further clarify the binding of complexes to G-quadruplex DNA [38]. The results of the fluorescence titration for these complexes with DNA are shown in Figure 4. Both of the complexes displayed a weakly emissive photoluminescence around 534 nm. The addition of HTG21-quadruplex resulted in an increase in emission intensity. It is worth noting that the increasing extent of the fluorescence intensity of complex** 1** shows stronger ability to bind DNA than complex** 2**.

**Figure 5:**
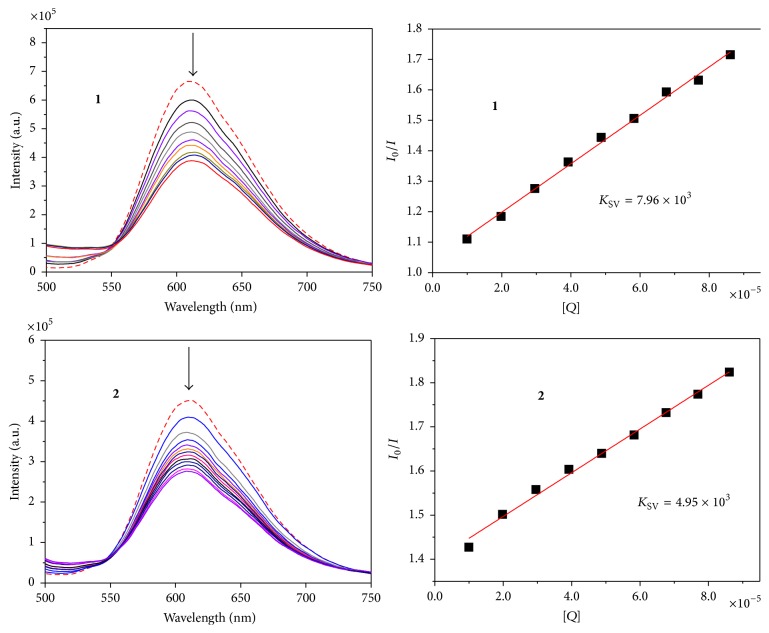
Fluorescence emission spectra of EthBr bound with G4-DNA ([DNA]/[EthBr] = 10 : 1) in the absence (- - - -) and presence (—) of increasing amounts of complexes** 1** and** 2** with [complex]/[EthBr] ratios ranging from 0 : 1 to 10 : 1, excited at 453 nm.

For further proof of intercalation, an ethidium bromide (EthBr) competitive binding study was undertaken. Ethidium bromide (EtBr) is a very fluorescent dye which has been shown to intercalate with nucleotides. EtBr showed characteristic fluorescent emission around 610 nm when it bound to DNA and EtBr fluorescence emission exhibited quenching when complexes** 1** and** 2** were added slightly, indicating that the intercalated modes between the base pairs of DNA and the compounds exist just similar to EtBr. The quenching constant (*K*_SV_) for complexes** 1** and** 2** was calculated to be 7.96 × 10^3^ and 4.95 × 10^3^, respectively, as shown in Figure 5. Results from these studies further confirm the ability to bind or stabilize G-quadruplex DNA.

## 4. Conclusion

In summary, we first synthesized and characterized Fe^II^ and Co^II^ complexes of natural product tryptanthrin and their G-quadruplex binding properties. Both complexes were found to display significant interaction with G-quadruplex. This study may potentially serve as the basis of future rational design of metal-based drugs from natural products that target the G-quadruplex. Consequently, future biological activities and the structure-activity relationships studies will be investigated.

## Figures and Tables

**Table 1:** The X-ray crystal data collection for complexes **1** and **2**.

Identification code	**1**	**2**
Empirical formula	C_60_H_32_FeN_8_O_8_	C_30_H_16_Co_0.5_N_4_O_4_·CH_3_OH
Formula weight	1016.79	541.97
Temperature/K	122(3)	296.15
Crystal system	Monoclinic	Monoclinic
Space group	C2/c	C2/c
*a*/Å, *b*/Å, *c*/Å	22.1273(20), 16.2600(9), 15.7165(14)	22.010(18), 18.811(16), 15.132(12)
*α*/°, *β*/°, *γ*/°	90.00, 120.361(12), 90.00	90.00, 117.842(8), 90.00
Volume/Å^3^	4879.1(7)	5540(8)
*Z*	4	8
*ρ*_calc_/mg mm^−3^	1.384	1.300
*μ*/mm^−1^	0.373	0.373
*F*(000)	2088	2236
Crystal size/mm^3^	0.30 × 0.15 × 0.08	0.35 × 0.25 × 0.11
2Θ range for data collection	5.82 to 50.24°	3.02 to 50.06°
Index ranges	−26 ≤ *h* ≤ 26, −19 ≤ *k* ≤ 18, −18 ≤ *l* ≤ 18	−26 ≤ *h* ≤ 26, −22 ≤ *k* ≤ 22, −17 ≤ *l* ≤ 18
Reflections collected	11653	19705
Independent reflections	4357 [*R*(int) = 0.0739]	4867 [*R*(int) = 0.1359]
Data/restraints/parameters	4357/0/340	4867/0/378
Goodness-of-fit on *F* ^2^	1.021	1.633
Final *R* indexes [*I* > 2*σ* (*I*)]	*R*_1_ = 0.0724, *wR*_2_ = 0.1286	*R*_1_ = 0.1712, *wR*_2_ = 0.4443
Final *R* indexes (all data)	*R*_1_ = 0.1345, *wR*_2_ = 0.1555	*R*_1_ = 0.2737, *wR*_2_ = 0.5060
Largest diff. peak/hole/*e* Å^−3^	0.559/−0.541	0.803/−0.997
